# A Pilot Study on the Ocular Effects of Colchicine in Children with Familial Mediterranean Fever

**DOI:** 10.3390/medicina62101885

**Published:** 2026-09-29

**Authors:** Berna Yüce, Tuba Çolak Erol, Mehmet Erol, Atılım Armağan Demirtaş, Esat Çınar, Belde Kasap Demir, Kayı Eliaçık

**Affiliations:** 1 Department of Ophthalmology, Izmir Faculty of Medicine, University of Health Sciences, 35540 Izmir, Türkiye; 2 Department of Ophthalmology, Izmir City Hospital, 35540 Izmir, Türkiye; 3 Department of Ophthalmology, Bazekol Hospital, 35620 İzmir, Türkiye; 4 Department of Pediatric Rheumatology, Faculty of Medicine, Izmir Kâtip Celebi University, 35620 Izmir, Türkiye; 5 Department of Pediatric Rheumatology, Izmir City Hospital, 35540 Izmir, Türkiye; 6 Department of Pediatric Diseases, Tepecik Training and Research Hospital, 35020 Izmir, Türkiye; 7 Department of Pediatric Diseases, Izmir Faculty of Medicine, University of Health Sciences, 35540 Izmir, Türkiye

**Keywords:** anterior chamber depth, axial length, colchicine, inflammation, keratometry, myopia

## Abstract

*Background and Objectives:* Myopia is a worldwide healthcare problem, particularly in the young population. Inflammation, alongside genetic and environmental factors, may contribute to the onset and progression of myopia. This pilot study explored whether long-term colchicine therapy is associated with ocular biometric and refractive differences in pediatric patients with Familial Mediterranean Fever (FMF). *Materials and Methods:* In this cross-sectional study, ocular biometric and refractive parameters were compared between FMF patients receiving colchicine for at least one year and age-matched healthy controls, including refraction, keratometry, anterior chamber depth (ACD), axial length (AL), and ganglion cell complex (GCC). *Results:* Compared with controls, the colchicine group showed a smaller refractive error (−0.46 ± 1.06 vs. −1.48 ± 1.93 D) and shallower ACD (3.29 ± 0.35 vs. 3.73 ± 0.31 mm); both remained significant after Holm-Bonferroni correction (corrected *p* = 0.041 and *p* < 0.001). Keratometry was also lower in the colchicine group but did not remain significant after correction (corrected *p* = 0.194). AL and GCC did not differ significantly between groups. *Conclusions:* Colchicine-treated FMF patients showed a less myopic profile and shallower anterior chamber depth than controls. Given the cross-sectional design and the inseparability of FMF status from colchicine treatment, these findings should be considered preliminary and hypothetical rather than evidence of a plausible protective effect, highlighting the need for longitudinal studies to clarify this potential association.

## 1. Introduction

Myopia is a refractive error in which light is focused anterior to the retina rather than directly on it [1]. In most cases, myopia is related to axial growth of the eyeball and less to changes in lenticular or corneal curvature. In recent years, the incidence of myopia has been increasing, especially among the young population. This has led to the emergence of the term ‘myopia pandemic’. It is projected that 50% of the world’s population will be myopic by 2050. Many factors, such as genetic and environmental influences, have been attributed to the onset of myopia. Furthermore, family history, close near-work distance, and reduced outdoor activity have been reported to be related to the onset or progression of myopia. However, the complete mechanism of myopia is not yet fully understood [2].

The retina is thought to act as a sensorimotor guide that analyzes image blur and uses signaling pathways to promote or inhibit scleral and choroidal growth during emmetropization of the eye [3].

Familial Mediterranean Fever (FMF) is a common, hereditary auto-inflammatory disease, characterized by recurrent fever and serosal inflammation associated with an increased acute-phase response. Autosomal recessive mutations in the Mediterranean fever (MEFV) gene, located on chromosome 16p13.3, lead to a decreased activation threshold for the pyrin inflammasome. Pyrin is a key protein in the NOD-like receptor protein 3 (NLRP3) inflammasome complex. In healthy individuals, the pyrin inflammasome is suppressed by RhoA-activated serine-threonine kinases (PKN1 and PKN2). Pyrin regulates the secretion of proinflammatory cytokines like interleukin (IL)-1β, IL-6, IL-18 and controls the immune response and cytoskeletal signaling. In the setting of mutant MEFV, caspase-1 cleaves pyrin, causing excessive release of inflammatory mediators. Overproduction of these cytokines triggers the release of inflammation-related proteins, such as amyloid and fibrinogen [4,5,6].

Colchicine, derived from Colchicum autumnale, has been the gold-standard treatment for FMF for decades. It reduces the number of attacks and helps to prevent amyloidosis, which is a devastating complication of FMF. Colchicine is a lipid-soluble alkaloid that acts on inflammatory cells. Colchicine targets leukocytes, neutrophils and macrophages, which are associated with inflammation. By blocking NLRP3 complex formation, colchicine reduces levels of IL-1β, IFN-γ, IL-6, and IL-18, and inhibits the action of these proinflammatory cytokines [5,6,7] (Figure 1).

Although colchicine is widely used for the treatment of FMF and gout, numerous studies have explored its potential use as an anti-inflammatory agent in conditions such as heart failure, metabolic syndrome, and diabetes, which have been linked to chronic inflammation [7].

In vivo studies have demonstrated that certain biological and chemical factors may be related to the onset of myopia. Understanding the molecular mechanisms of myopia may help define new therapeutic strategies.

This study aimed to evaluate the effect of colchicine on refractive status and ocular biometric parameters in the eyes of children with FMF.

## 2. Material and Methods

A total of 17 pediatric patients (10–18 years old) with genetically and clinically confirmed FMF who were receiving colchicine therapy were enrolled in the study, along with 28 age-matched healthy controls. FMF patients were selected from those who had been followed at the Pediatric Rheumatology Clinic for at least one year. Healthy controls were selected from individuals who presented to the Ophthalmology Outpatient Clinic for routine ophthalmic examination. The inclusion criterion for both groups was being 10 years of age or older. The inclusion criterion for FMF patients were being in the remission phase of the disease and receiving colchicine treatment for at least one year.

Exclusion criteria for both groups were parental myopia greater than 6 diopters; any history of ocular trauma, intraocular surgery, neuro-ophthalmological disease, uveitis, strabismus, congenital cataract, congenital glaucoma, corneal, retinal, or vitreous pathology; and any systemic disease that could cause visual impairment.

The right eye parameters of each individual were selected for analysis. Uncorrected (UDVA) and best corrected distance visual acuity (BCDVA), refractive error (RE) and keratometry (K) measured by autorefractokeratometry (Canon RK-F2, Kawasaki, Japan), axial length (AL), anterior chamber depth (ACD) and white-to-white distance (WWD) measured with optical biometry (Nidek AL-Scan, Nidek Co., Gamagori, Japan) and ganglion cell complex (GCC) thickness values obtained from the spectral-domain-OCT (SD-OCT) images (Heidelberg OCT Spectralis, version 1.7.0.0, Heidelberg Engineering, Heidelberg, Germany) were evaluated for each individual. Slit-lamp biomicroscopy and fundus examination were performed for all participants. Non-cycloplegic refraction was preferred in order to minimize the potential influence of cycloplegic agents on anterior segment parameters, most particularly the ACD.

All statistical analyses were performed using IBM SPSS Statistics for Windows, version 26.0 (IBM Corp., Armonk, NY, USA). Categorical variables were expressed as frequencies and percentages (*n*, %) and assessed by Chi-Square test. Descriptive statistics are presented as the mean ± standard deviation (Mean ± SD) for continuous variables with a normal distribution. For the comparison of continuous ophthalmological parameters between the study groups, the parametric Student’s *t*-test or the non-parametric Mann–Whitney U test was used, as appropriate. If the Shapiro–Wilk test indicated a significant deviation from normality, the Mann–Whitney U test was used for between-group comparisons. Spearman’s correlation analysis was used to examine the relationship between the parameters. To control for type I error inflation across multiple comparisons, we applied the Holm–Bonferroni step-down correction. A two-tailed *p*-value of <0.05 was considered statistically significant for all analyses.

The study was conducted in accordance with the Declaration of Helsinki and the protocol was approved by the Institutional Review Board and Ethics Committee (decision number: 2023/04-22) on 3 May 2023. Informed consent was obtained from all legal guardians of the pediatric patients involved in the study.

## 3. Results

The mean age was 13.12 ± 2.26 (10–17) years in the colchicine group and 14.36 ± 2.39 (10–17) years in the control group, indicating no significant difference (*p* = 0.095, Mann–Whitney U Test). The female/male ratio was 9/8 in the colchicine group and 18/10 in the healthy controls. The analysis showed no statistically significant difference in gender distribution between the groups (*p* = 0.451, Chi-Squared Test).

The mean age of FMF onset was 5.24 ± 2.87 (0.6–11) years and the mean duration of colchicine administration was 7.79 ± 3.0 (2.5–13.5) years. All patients had been continuously treated throughout the reported period. The mean dose of colchicine was 1.25 ± 0.25 (1–1.5) mg in FMF patients.

The colchicine group had a mean refraction of −0.46 ± 1.06 D (range: −4.00 to 0.50), while the control group had a mean refraction of −1.48 ± 1.93 D (range: −8.25 to 2.00). The analysis revealed that participants in the control group exhibited a more myopic status than those in the colchicine group and there was a statistically significant difference between the two groups (U = 368.5, *p* = 0.009, Mann–Whitney U test).

The mean keratometry value (K_mean_) was 43.09 ± 1.35 D (range: 40.25–45.00) in the colchicine group compared to 44.02 ± 1.45 D (range: 41.17–46.50) in the control group. The statistical analysis revealed a significant difference between the two groups (*p* = 0.039, independent samples *t*-test). These findings indicate that the control group had significantly higher K_mean_ values than the colchicine group.

The mean ACD was 3.29 ± 0.35 mm (range: 2.65–3.87) in the colchicine group, compared to 3.73 ± 0.31 mm (range: 3.15–4.32) in the control group (*p* < 0.0001, independent samples *t*-test). These results demonstrated that the colchicine group had significantly shallower anterior chamber depths than the control group.

The mean AL was 23.48 ± 0.98 mm (range: 22.35–26.31) in the colchicine group, compared to 23.69 ± 1.16 mm (range: 21.56–26.29) in the control group. Statistical analysis revealed no significant difference between the two groups (U = 204.0, *p* = 0.433, Mann–Whitney U test).

The mean WWD was 12.35 ± 0.40 mm (range: 11.69–13.30) in the colchicine group, compared to 12.10 ± 0.51 mm (range: 11.20–12.96) in the control group. The statistical analysis revealed no significant difference between the two groups (*p* = 0.089, independent samples t-test).

The mean GCC thickness was 109.41 ± 5.28 μm (range: 101.88–120.75) in the colchicine group, compared to 107.99 ± 5.34 μm (range: 97.75–124.25) in the control group. There was no statistically significant difference between the groups in terms of GCC thickness (U = 274.0, *p* = 0.406, Mann–Whitney U test).

After Holm-Bonferroni correction, the differences in ACD (corrected *p* = 0.0004) and refraction (corrected *p* = 0.041) remained statistically significant, whereas the difference in Kmean was no longer statistically significant (corrected *p* = 0.194). These findings should therefore be interpreted with caution, as discussed in the Limitations section (Table 1).

No statistically significant correlations were found between treatment duration and refraction (rho = −0.328, *p* = 0.198), K_mean_ (rho = −0.249, *p* = 0.335), AL (rho = 0.207, *p* = 0.424), ACD (rho = −0.400, *p* = 0.112), WWD (rho = −0.230, *p* = 0.375), or GCC thickness (rho = −0.374, *p* = 0.139). Similarly, colchicine dosage showed no statistically significant correlation with refraction (rho = −0.234, *p* = 0.365), K_mean_ (rho = 0.000, *p* = 1.000), AL (rho = 0.087, *p* = 0.741), ACD (rho = 0.111, *p* = 0.670), WWD (rho = 0.248, *p* = 0.338), or GCC thickness (rho = −0.062, *p* = 0.813) (Table 2).

## 4. Discussion

Myopia is a public health issue that affects billions of people globally and causes visual impairment, especially among young people. Increased screen time, decreased outdoor activity, and reduced daylight exposure have been found to be associated with myopia [8].

Extensive research is currently underway on the treatment and control of myopia progression. Various algorithms have been proposed for myopia management, and intensive studies are being conducted on therapeutic modalities such as pharmacological eye drops, specially designed spectacle lenses, and contact lenses. Pharmacological eye drops, particularly low-dose atropine, act by modulating muscarinic receptor activity, thereby reducing scleral remodeling and slowing axial elongation [9,10]. Specially designed spectacle lenses, such as defocus-incorporated multiple segments (DIMS) or highly aspherical lenslets (HAL), create peripheral myopic defocus to alter retinal signaling and inhibit excessive eye growth [11,12]. Contact lenses, including multifocal designs and orthokeratology, induce controlled peripheral defocus or reshape corneal curvature, ultimately reducing axial elongation [13,14]. Together, these modalities represent complementary strategies targeting both optical and cellular mechanisms, and ongoing clinical trials continue to refine their efficacy, safety, and long-term impact on myopia progression.

Myopia is primarily associated with axial elongation, referred to as “axial myopia,” or with increased corneal or lenticular refractive power, referred to as “refractive myopia” [1]. The most common form, axial myopia, arises from scleral extracellular matrix remodeling, which results in abnormal globe elasticity. The sclera primarily consists of Collagen types I, III, and IV. Collagen I constitutes almost 50–70% of the scleral collagen composition. It has been demonstrated that matrix metalloproteinases (MMPs) promote degradation of scleral Collagen I, resulting in reduced Collagen I levels and extracellular matrix remodeling in myopic eyes [15].

Inflammation has been shown to play a role in the pathogenesis of numerous systemic and ocular diseases. Within the eye, specific inflammatory cytokines—including TNF-α, IL-1β, IL-6, IL-8, IL-17 and IL-18—have been identified as key mediators of intraocular inflammation, contributing to conditions such as allergic conjunctivitis, uveitis, and age-related macular degeneration, through their effects on the retinal endothelium and the blood-retinal barrier [16,17].

The relationship between myopia and inflammation has been increasingly recognized in recent years. Earlier studies demonstrated elevated levels of interleukins such as IL-1, IL-6, and IL-17 in patients with high myopia, suggesting that immune mediators may contribute to scleral remodeling and axial elongation. These findings suggest that inflammatory pathways may play a causal role in myopia progression [18,19,20,21,22,23].

The inflammasome represents a critical component of the innate immune system, functioning as a multimeric protein complex that assembles in the cytosol upon sensing pathogen-associated molecular patterns (PAMPs), such as microbial toxins and viral RNA, or danger-associated molecular patterns (DAMPs), such as uric acid crystals, cholesterol crystals, and reactive oxygen species (ROS). Among the various inflammasome types, the NLRP3 inflammasome has been the most extensively studied, owing to its ability to respond to the widest array of stimuli. Its activation requires two sequential steps: a priming signal, typically mediated through NF-κB signaling, which upregulates NLRP3 and pro-IL-1β expression and enables NLRP3 via deubiquitination, and a second, distinct activating signal, such as potassium efflux, mitochondrial reactive oxygen species generation, or lysosomal destabilization, which triggers oligomerization of NLRP3 with the adaptor protein ASC and pro-caspase-1. This assembly leads to autoproteolytic activation of caspase-1, which subsequently cleaves pro-IL-1β and pro-IL-18 into their biologically active forms and induces pyroptosis, an inflammatory form of programmed cell death. Dysregulated NLRP3 inflammasome activation has been implicated in a broad spectrum of chronic inflammatory and degenerative conditions, including neurodegenerative diseases and metabolic disorders, where it contributes to tissue damage through sustained IL-1β production and downstream inflammatory cascades [24,25]. It has also previously been demonstrated that NLRP3 expression is increased in various ocular pathologies, such as diabetic retinopathy, age-related macular degeneration, and glaucoma [26,27,28].

In their experimental study examining wild-type and NLRP3-/- mouse eyes under blank (untreated), 2-week masking, 4-week masking, and 4-week masking followed by 1-week unmasking conditions, Chen et al. [29] found no change in refraction or axial length (AL) relative to baseline in the blank group of wild-type mice. However, the researchers observed a pronounced myopic shift (5.30 vs. 9.20 D) and AL elongation (3070 vs. 2945 µm) in wild-type mice subjected to 4 weeks of masking. In NLRP3-/- mice, although there was some increase in myopia following 4 weeks of masking (7.33 vs. 9.15 D), this increase was found to be significantly less than that observed in wild-type mice (5.30 vs. 7.33 D). No significant change in AL was observed within this group. The authors also observed that removal of the mask for 1 week after 4 weeks of masking led to a marked reversal of the myopic shift (from 9.90 to 5.30 D). IL-1β and MMP-2 levels were found to be higher in the masked eyes of wild-type mice compared to control eyes, whereas collagen I levels showed the opposite trend, reaching their lowest point in the 4-week masking group. They also demonstrated that IL-18 levels were elevated in all masked eyes. In light of these results, the authors suggest that the NLRP3 pathway is strongly associated with myopia progression. In this study, which was one of the first to demonstrate a relationship between the NLRP3 pathway and myopia, the elevation of IL-18 levels even in NLRP3-/- eyes, along with the failure to completely prevent myopia, was explained by the idea that myopia is a multifactorial pathology and that pathways other than NLRP3 may also be involved.

In our study, we demonstrated that FMF patients receiving colchicine treatment had a less myopic refractive status, shallower anterior chamber depth, and lower keratometric values. As is well established, the NLRP3–caspase-1–IL-1β axis mediates chronic low-grade inflammation and tissue remodeling across multiple organ systems. It is plausible that similar inflammasome-driven mechanisms may have contributed to the ocular changes observed in our study, suggesting a potential link between systemic inflammatory suppression—such as that achieved with colchicine—and the altered anterior segment and refractive parameters in the colchicine-treated group compared to controls.

Chen et al. [15] established an experimental form-deprivation myopia model in mice and evaluated the effect of intraperitoneally administered MCC950, a selective NLRP3 inflammasome inhibitor, on axial elongation and myopic shift. The authors demonstrated that MCC950 injection reduced axial elongation and myopia progression, accompanied by downregulation of NLRP3, caspase-1, IL-1β, IL-18, and MMP-2, and preservation of Collagen-1 expression in the sclera of form-deprived mice, but did not induce any changes in non-deprived mice. These findings support the hypothesis that inflammatory signaling through the NLRP3–MMP-2–Collagen-1 pathway contributes to scleral extracellular matrix remodeling and axial elongation in myopia. Their work provides preliminary experimental evidence that targeting NLRP3 inflammasome activity may represent a novel strategy for controlling myopia progression.

In a recent study published by Xiao et al. [30], the authors induced form-deprivation myopia in two different groups of mice, wild-type and C6-knockout, and compared the myopic shift and axial elongation values between the two groups following 4 weeks of form deprivation. The researchers reported that the C6-knockout group exhibited less myopic shift (−2.28 D vs. −5.40 D) and less axial elongation (0.043 mm vs. 0.083 mm) compared to the wild-type group. Furthermore, through immunohistochemical analyses, the authors found that levels of C5b-9, NLRP3, caspase-1, IL-1β, and MMP-2 were significantly elevated in wild-type mice following form deprivation compared to the C6-knockout group. These findings support the notion that the complement cascade, and particularly NLRP3-related inflammatory processes, may play a role in the development of myopia by directly affecting scleral biomechanics. The authors suggested that inhibitors targeting inflammatory pathways such as C5b-9 could represent a potential therapeutic target in the treatment of myopia. The less myopic refraction and shallower anterior chamber depth observed in our colchicine-treated group, in the absence of a corresponding difference in axial length, are consistent with—though not direct evidence for—a role of the inflammatory pathways demonstrated by Xiao et al. It should be emphasized that our cross-sectional design did not include measurements of NLRP3, complement, or other inflammatory biomarkers, and that the proposed mechanistic link remains a hypothesis to be tested in future studies rather than a conclusion supported by the present data.

Beyond its role in scleral remodeling, NLRP3 inflammasome activation has recently been directly implicated in corneal pathology under environmental stress conditions. Ge et al. [31] showed that exposing mouse corneas and cultured human corneal epithelial cells to blue light compromised epithelial integrity and slowed the recovery process following injury, which were mediated through activation of the NLRP3–ASC–caspase-1 axis and subsequent IL-1β and IL-18 production. In this model, pharmacological inhibition of NLRP3 with MCC950 attenuated reactive oxygen species (ROS) accumulation and partially restored mitochondrial membrane potential, in addition to suppressing inflammasome activation itself. This is consistent with the broader concept, described by Abais et al. [32], that NLRP3 inflammasome activity and ROS production may reinforce one another through a positive feedback loop rather than following a strictly unidirectional trigger-response relationship. A possible ROS–NLRP3 interplay may underlie, at least in part, the anterior segment changes observed in our study, although this remains a hypothesis that requires direct testing in future investigations.

Colchicine is an alkaloid extracted from the autumn crocus plant. It has been used since ancient times for the treatment of joint pain and swelling. It is currently widely used for gout, FMF, Behçet’s disease, pericarditis, osteoarthritis, and coronary artery disease. It acts by binding tubulin and preventing microtubule formation, thereby disrupting the internal cytoskeleton of the cell. Blockade of microtubule formation inhibits neutrophil activation, degranulation, and migration to sites of inflammation, and decreases cytokine release, reactive oxygen species production, and NLRP3 inflammasome formation. Blockage of the NLRP3 inflammasome, which is a complex normally responsible for activating caspase-1 and driving the subsequent processing and release of IL-1β and IL-18, results in the suppression of the release of these inflammatory molecules. Thus, colchicine suppresses the inflammatory responses [33].

Colchicine is the standard treatment for FMF, and its safety profile at maintenance doses is well characterized. The most common adverse effects occur early in treatment and consist of gastrointestinal complaints such as nausea and diarrhea. Data collected over decades show that long-term colchicine use does not increase the risk of malignancy, infection, cytopenia, myotoxicity, gastrointestinal bleeding, or peripheral neuropathy. Serious toxicity is rare and mainly occurs with substantial overdose or in patients with advanced renal or hepatic impairment, where reduced drug clearance allows serum concentrations to exceed established safety thresholds [34].

In our study, the colchicine-treated FMF group showed a significantly shallower anterior chamber depth and a less myopic refractive profile compared with controls, differences that remained statistically significant after correction for multiple comparisons (Holm-Bonferroni method). The colchicine group also showed a flatter cornea compared with controls, without a corresponding difference in axial length; however, this difference in keratometry did not remain robust after correction for multiple comparisons and should therefore be interpreted with caution. The keratometric and anterior chamber depth findings in our study occurred in the absence of a significant difference in axial length between groups. This pattern differs from the classic scleral remodeling mechanism described in the myopia literature, in which chronic scleral inflammasome (NLRP3–caspase-1) activation drives fibroblast pyroptosis, ECM degradation, and axial elongation. The differences we observed may therefore reflect an anterior segment process, although this remains speculative and requires further investigation.

The mechanism responsible for the reduced anterior chamber depth observed in colchicine-treated patients in our study remains unclear. Previous studies suggested that colchicine can reach ocular tissues directly. Leibovitch et al. [35] showed that colchicine is detectable in the tear fluid of FMF patients on long-term treatment, indicating that the drug can also reach periocular tissue. This is consistent with reports of delayed corneal wound healing in colchicine-treated patients, which is linked to the drug’s effects on cell division and collagen turnover. However, there is no study assessing the effect of colchicine on anterior chamber biomechanics. Our finding of a reduced anterior chamber depth should therefore be interpreted with caution, and further studies are needed to clarify whether colchicine has a direct effect on anterior chamber structure.

Although it is not fully elucidated which cells contribute to emmetropization of the eye and are therefore responsible for the onset of myopia, dopaminergic amacrine cells may play a role. It has been reported that dopamine agonists, such as apomorphine, cause a decrease in myopia, whereas dopamine antagonists cause an increase. It has been shown that amacrine cells affect both the induction and inhibition of myopia. Amacrine cells modulate the signals transmitted to retinal ganglion cells. It has also been suggested that retinal ganglion cells respond to the focus/defocus of the retinal image, thereby modulating ocular growth and refractive status [3].

Nevertheless, our study revealed no significant difference in GCC thickness between the more myopic control group and the less myopic colchicine group. Similar to our study, Alim et al. [36] reported no significant changes in either RNFL or ganglion cell–inner plexiform layer thickness in adult-onset FMF patients, independent of colchicine use or disease severity. Taken together, these findings suggest that oral colchicine therapy does not appear to affect retinal ganglion cell integrity in FMF.

Fischer et al. [37] reported in an experimental chick model that intravitreal colchicine accelerates ocular growth and induces myopia through ganglion and amacrine cell loss. Conversely, our findings suggest that systemic therapeutic doses of colchicine in FMF patients are not associated with retinal ganglion cell damage. Whether this reflects an effect on inflammatory pathways relevant to myopia cannot be determined from our cross-sectional study and should be regarded as speculative.

This study has several important limitations. First, the small sample size and cross-sectional design represent major constraints. The cross-sectional nature does not permit the assessment of longitudinal refractive change or axial elongation, and therefore cannot establish whether colchicine influences myopia progression. Second, the colchicine-treated group consisted exclusively of children with FMF, whereas the control group comprised healthy children; thus, FMF status, systemic inflammation, and colchicine treatment are inherently confounded in our design, and the observed differences cannot be attributed specifically to colchicine, even though patients experiencing active FMF attacks were not enrolled to the study. Other potential confounders—including parental myopia, socioeconomic factors, near-work habits, and outdoor exposure—were not systematically recorded and could not be adjusted for. Third, non-cycloplegic refraction was used to avoid cycloplegia-related changes in anterior chamber depth; however, accommodation may bias refraction toward more myopic values in children. Fourth, multiple ocular parameters and correlation analyses were tested. We applied the Holm-Bonferroni step-down correction for the between-group comparisons, to reduce the risk of type I error. Given these limitations, the findings in our study should be interpreted with caution. Larger, longitudinal, prospective studies incorporating cycloplegic refraction, serial axial-length measurement, assessment of FMF severity, and adjustment for known myopia-related confounders are needed to clarify whether colchicine has any effect on myopia development or progression.

## 5. Conclusions

Treatment modalities targeting inflammatory pathways may have a potential role in myopia control. Although our study was conducted in a small patient group as a pilot analysis, it represents, to our knowledge, the first human study to examine ocular refractive and biometric parameters in colchicine-treated FMF patients. Our cross-sectional findings demonstrate an association between colchicine-treated FMF status and a shallower anterior chamber depth and a less myopic refractive profile—differences that remained statistically significant after correction for multiple comparisons—along with flatter keratometry, a finding that did not remain robust after correction for multiple comparisons and should be interpreted with caution; none of these findings establish a protective or causal effect of colchicine on the development or progression of myopia. Larger, longitudinal, prospective studies in broader patient populations, incorporating cycloplegic refraction, serial axial-length measurement, and adjustment for relevant confounders, are warranted to determine whether colchicine has a genuine protective effect on myopia.

## Figures and Tables

**Figure 1:**
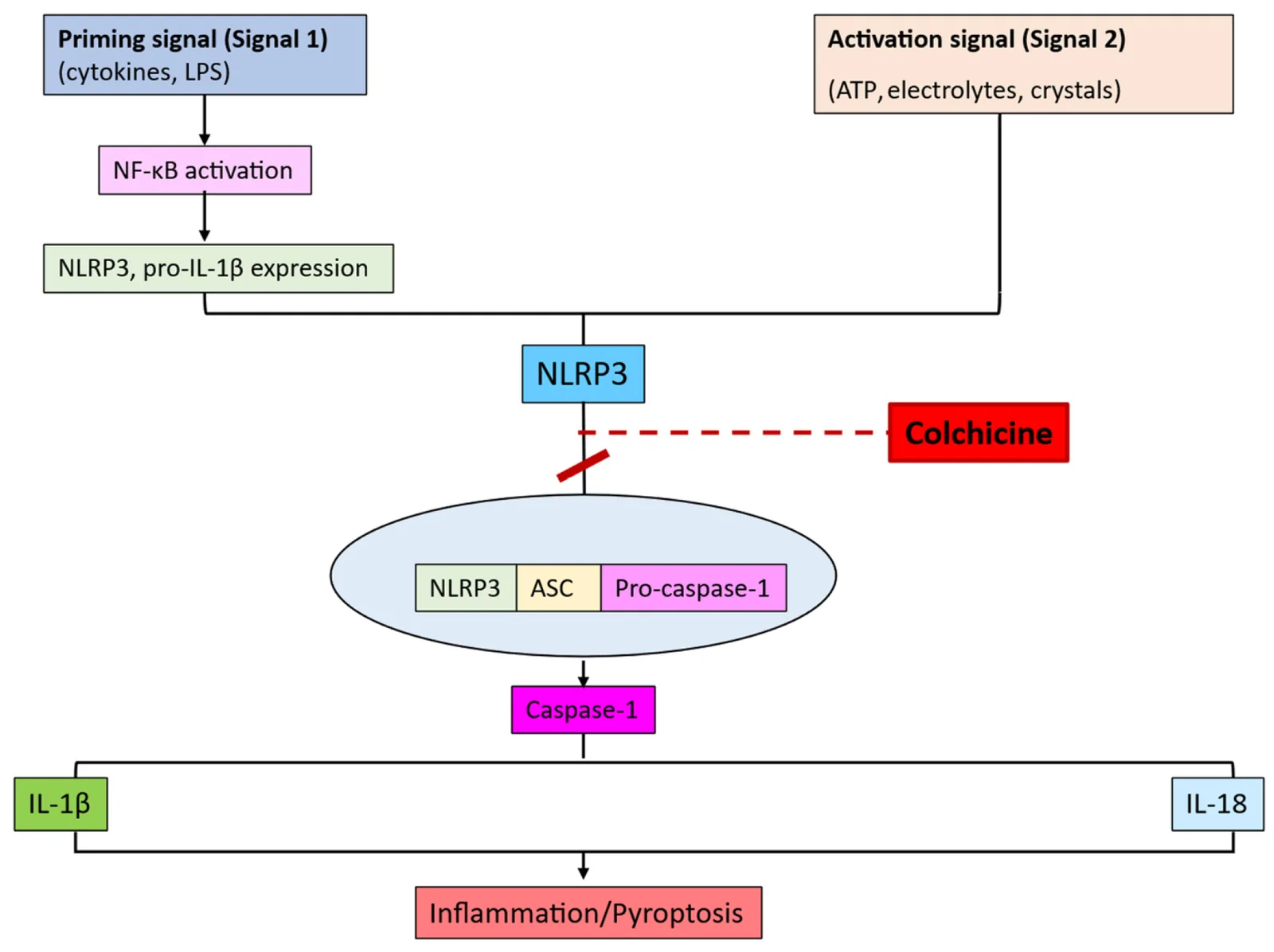
Activation of the NLRP3 inflammasome requires two signals. Signal 1 (priming), provided by stimuli such as cytokines and LPS, activates NF-κB signaling, which upregulates the expression of NLRP3 and pro-IL-1β. Signal 2 (activation), provided by stimuli such as ATP, electrolyte flux, and crystals, acts together with Signal 1 to trigger NLRP3 activation. Colchicine inhibits this step, blocking downstream assembly of the NLRP3 inflammasome complex (NLRP3, ASC, and pro-caspase-1). Under normal conditions, inflammasome assembly activates caspase-1, which cleaves pro-IL-1β and pro-IL-18 into their mature, active forms (IL-1β and IL-18), leading to inflammation and pyroptosis. (NF-κB, nuclear factor kappa B; LPS, lipopolysaccharide; NLRP3, NOD-, LRR-, and pyrin domain-containing protein 3; ASC, apoptosis-associated speck-like protein containing a CARD; IL, interleukin).

**Table 1:** Comparison of Demographic and Ophthalmological Parameters Between Colchicine and Control Groups.

Parameter	Colchicine Group (n = 17)	Control Group (n = 28)	Raw *p*-Value	Adjusted *p*-Value (Holm-Bonferroni)
Age (years)	13.12 ± 2.26	14.36 ± 2.39	0.095 ^a^	0.356
Gender, F/M, n (%)	9/8 (52.9/47.1)	18/10 (64.3/35.7)	0.451 ^b^	-
Refraction (D)	−0.46 ± 1.06	−1.48 ± 1.93	**0.009** ^a^	**0.041**
K_mean_ (D)	43.09 ± 1.35	44.02 ± 1.45	**0.039** ^c^	0.194
ACD (mm)	3.29 ± 0.35	3.73 ± 0.31	**<0.001** ^c^	**<0.001**
AL (mm)	23.48 ± 0.98	23.69 ± 1.16	0.433 ^a^	0.811
WWD (mm)	12.35 ± 0.40	12.10 ± 0.51	0.089 ^c^	0.356
GCC (μm)	109.41 ± 5.28	107.99 ± 5.34	0.406 ^a^	0.811

^a^: Mann–Whitney U Test. ^b^: Chi-Squared Test. ^c^: Independent Samples *t*-test. Statistically significant *p*-values are shown in bold. Abbreviations: D, diopters; ACD, anterior chamber depth; AL, axial length; WWD, white-to-white distance; GCC, ganglion cell complex.

**Table 2:** Spearman’s Correlation Between Colchicine Treatment Duration/Dosage and Ocular Parameters in the Colchicine Group.

Parameter	Treatment Duration		Colchicine Dosage	
	rho	*p*-Value	rho	*p*-Value
Refraction (D)	−0.328	0.198	−0.234	0.365
K_mean_ (D)	−0.249	0.335	0.000	1.000
AL (mm)	0.207	0.424	0.087	0.741
ACD (mm)	−0.400	0.112	0.111	0.670
WWD (mm)	−0.230	0.375	0.248	0.338
GCC (μm)	−0.374	0.139	−0.062	0.813

Spearman’s rank correlation coefficient (rho) was used to assess the relationship between treatment duration, colchicine dosage, and ocular parameters within the colchicine group. A two-tailed *p*-value < 0.05 was considered statistically significant. No statistically significant correlations were observed. Abbreviations: D, diopters; AL, axial length; ACD, anterior chamber depth; WWD, white-to-white distance; GCC, ganglion cell complex.

## Data Availability

The raw data supporting the conclusions of this article will be made available by the authors on request; the data are not publicly available due to ethical restrictions.
